# RANKL Signaling and Osteoclastogenesis Is Negatively Regulated by Cardamonin

**DOI:** 10.1371/journal.pone.0064118

**Published:** 2013-05-17

**Authors:** Bokyung Sung, Sahdeo Prasad, Vivek R. Yadav, Subash C. Gupta, Simone Reuter, Norio Yamamoto, Akira Murakami, Bharat B. Aggarwal

**Affiliations:** 1 Cytokine Research Laboratory, Department of Experimental Therapeutics, The University of Texas MD Anderson Cancer Center, Houston, Texas, United States of America; 2 Food Science Research Center, House Wellness Foods Corporation, Itami, Japan; 3 Division of Food Science and Biotechnology, Graduate School of Agriculture, Kyoto University, Kyoto, Japan; Virginia Commonwealth University, United States of America

## Abstract

Bone loss/resorption or osteoporosis is a disease that is accelerated with aging and age-associated chronic diseases such as cancer. Bone loss has been linked with human multiple myeloma, breast cancer, and prostate cancer and is usually treated with bisphosphonates, and recently approved denosumab, an antibody against receptor activator of NF-κB ligand (RANKL). Because of the numerous side effects of the currently available drugs, the search continues for safe and effective therapies for bone loss. RANKL, a member of the TNF superfamily, has emerged as a major mediator of bone loss via activation of osteoclastogenesis. We have identified cardamonin, a chalcone isolated from *Alpinia katsumadai* Hayata that can affect osteoclastogenesis through modulation of RANKL. We found that treatment of monocytes with cardamonin suppressed RANKL-induced NF-κB activation and this suppression correlated with inhibition of IκBα kinase and of phosphorylation and degradation of IκBα, an inhibitor of NF-κB. Furthermore, cardamonin also downregulated RANKL-induced phosphorylation of MAPK including ERK and p38 MAPK. Cardamonin suppressed the RANKL-induced differentiation of monocytes to osteoclasts in a dose-dependent and time-dependent manner. We also found that an inhibitor of NF-κB essential modulator (NEMO) blocked RANKL-induced osteoclastogenesis, indicating a direct link with NF-κB. Finally, osteoclastogenesis induced by human breast cancer cells or human multiple myeloma cells were completely suppressed by cardamonin. Collectively, our results indicate that cardamonin suppresses osteoclastogenesis induced by RANKL and tumor cells by suppressing activation of the NF-κB and MAPK pathway.

## Introduction

Bone undergoes constant turnover and is kept in balance (homeostasis) by osteoblasts (creating bone) and osteoclasts (destroying bone). The osteoclast is a unique bone-resorbing cell derived from cells of monocyte-macrophage lineage. Osteoclastogenesis comprises many stages, including commitment, differentiation, multinucleation, and activation of immature osteoclasts. Within the bone microenvironment, T and B lymphocytes, bone marrow stromal cells, macrophages, and osteoblasts all can produce cytokines that have an impact on osteoclastogenesis. A variety of both systemic hormones and cytokines regulate osteoclast differentiation and function [1].

Receptor activator of nuclear factor-κB ligand (RANKL), a member of the tumor necrosis factor (TNF) superfamily, has emerged as a major osteoclastogenic cytokine. Binding of RANKL to its receptor, RANK, on macrophages prompts them to assume the osteoclast phenotype [2], [3]. RANKL is expressed on the surface of osteoblastic/stromal cells and by various types of cancer cells, and is directly involved in the differentiation of monocyte macrophages into osteoclasts [4]. When RANKL binds to RANK, it undergoes trimerization and then binds to an adaptor molecule, TNF receptor-associated factor 6 (TRAF6), which then sequentially activates IκBα kinase (IKK), nuclear factor-κB (NF-κB), and nuclear factor of activated T cells, cytoplasm 1 (NFATc1), thus leading to osteoclastogenesis [5], [6].

Several types of cancer, both solid and hematopoietic, are deeply linked with the skeleton and cause an increase in osteoclast formation, either systemically, as in humoral hypercalcemia of malignancy, or locally, as in bone metastasis. Bone metastasis makes bone more fragile and leads to pathologic fractures and spinal compression. This osteolysis is associated with severe bone pain, which may be intractable. Bone metastasis represents a common cause of morbidity in patients with many types of cancer, occurring in as many as 70% of patients with advanced breast or prostate cancer and in about 15% to 30% of patients with lung, colon, kidney, thyroid, or stomach carcinoma [7], [8]. It is more common in patients with advanced multiple myeloma or breast, prostate, or lung cancer, as these tumors have a remarkable propensity to metastasize to the bone [9], [10].

RANKL has been shown to play a major role in bone metastasis [9], and thus is an important therapeutic target. Thus, agents that suppress RANKL signaling have potential for inhibition of osteoclastogenesis and bone metastasis. Previous studies have demonstrated that cardamonin ((2E)-1-(2,4-Dihydroxy-6-methoxyphenyl)-3-phenyl- 2-propen-1-one)), a chalcone isolated from grass cardamom (*Alpinia katsumadai* Hayata) and black cardamom (*Amomum subulatum*) can suppress NF-κB activation induced by a variety of agents [11], [12]. Because both RANKL and NF-κB pathway have been closely linked with osteoclastogenesis, we decided to investigate the effect of this chalcone on RANKL-induced NF-κB activation pathway and osteoclastogenesis. We found that cardamonin can suppress RANKL-induced NF-κB activation through inhibition of IKK and inhibits osteoclastogenesis induced by RANKL and by breast cancer and multiple myeloma cells.

## Materials and Methods

### Reagents

Cardamonin (synthesized and kindly provided by Dr. Norio Yamamoto) was prepared as 20 mM solution in dimethyl sulfoxide and then further diluted in cell culture medium. Dulbecco modified essential medium (DMEM)/F12, RPMI 1640, DMEM, fetal bovine serum, 0.4% trypan blue vital stain, and antibiotic-antimycotic mixture were obtained from Mediatech, Inc (Manassas, VA). Recombinant RANKL protein was kindly provided by Dr. Bryant Darnay of The University of Texas MD Anderson Cancer Center (Houston, TX). Antibodies against RANKL, IKKα, IKKβ and IκBα were purchased from Imgenex (San Diego, CA), while cell-permeable NF-κB essential modulator (NEMO; also called IKKγ)−binding domain peptide (NBP) were kind gifts from Imgenex. Antibodies against phospho-IκBα (Ser32/36), and phospho-IKKα (Ser176)/IKKβ (Ser177) were purchased from Cell Signaling Technology (Danvers, MA). Antibodies against c-Src, NFATc1, MMP-9, phospho-ERK1/2 (Thr202/Tyr204), ERK2, phospho-p38 (Tyr180/182) and p38 were obtained from Santa Cruz Biotechnology (Santa Cruz, CA). Goat anti-rabbit and goat anti-mouse horseradish peroxidase conjugates were purchased from Bio-Rad (Hercules, CA). β-actin antibody and leukocyte acid phosphatase kit (387-A) for tartrate-resistant acid phosphatase (TRAP) staining were purchased from Sigma-Aldrich (St. Louis, MO). Protein A/G-agarose beads were obtained from Thermo Scientific (Rockford, IL). [γ^32^P]ATP was purchased from MP Biomedicals (Solon, OH).

### Cell lines

RAW 264.7 (mouse macrophage) cells were kindly provided by Dr. Bryant Darnay. For these studies, we used a single clone (#28) that has been selected after limited dilution. RAW 264.7 cells were cultured in DMEM/F12 supplemented with 10% fetal bovine serum and antibiotics. This cell line is a well-established osteoclastogenic cell system that has been shown to express RANK and to differentiate into functional TRAP-positive osteoclasts when cultured with soluble RANKL [13]. Moreover, RANKL has been shown to activate NF-κB in RAW 264.7 cells [14]. MDA-MB-231 (human breast adenocarcinoma) and U266 (human multiple myeloma) cells were obtained from the American Type Culture Collection (Manassas, VA). MDA-MB-231 cells were cultured in DMEM and U266 cells in RPMI 1640 with 10% fetal bovine serum.

### Osteoclast differentiation assay

RAW 264.7 cells were cultured in 24-well plates at a density of 5×10^3^ per well and allowed to adhere overnight. The medium was then replaced, and the cells were treated with 5 nM RANKL for 5 days. All cells were subjected to TRAP staining using the leukocyte acid phosphatase kit. For co-culture experiments with cancer cells, RAW 264.7 cells were seeded at 5×10^3^ per well and allowed to adhere overnight. The following day, U266 or MDA-MB-231 cells, at 1×10^3^ per well, were added to the RAW 264.7 cells, treated with cardamonin, and co-cultured for 5 days before being subjected to TRAP staining.

### Electrophoretic mobility shift assay (EMSA) for NF-κB

Nuclear extracts were prepared and EMSA was carried out as described previously [15]. Briefly, equal amount of nuclear extracts from untreated and RANKL-treated cells were incubated with ^32^P-end-labeled 45-mer double-stranded NF-κB oligonucleotide (15 µg protein with 16 fmol DNA) from the HIV long terminal repeat, 5′-TTGTTACAAGGGACTTTCCGCTGGGGACTTTCCAGGGGGAGGCGTGG-3′ (underline indicates NF-κB–binding sites), for 30 min at 37°C, and the DNA-protein complex formed was separated from free oligonucleotide on 6.6% native polyacrylamide gels. The dried gels were visualized with a Storm820, and radioactive bands were quantified using a densitometer and Image Quant software (GE Healthcare, Piscataway, NJ).

### Western blot analysis

To determine the levels of protein expression in the cytoplasm and nucleus, we prepared extracts and fractionated them by sodium dodecyl sulfate polyacrylamide gel electrophoresis (SDS-PAGE). After electrophoresis, the proteins were electrotransferred to nitrocellulose membranes, blotted with relevant antibodies, and detected with a chemiluminescence reagent (GE Healthcare).

### IKK assay

To determine the effect of cardamonin on RANKL-induced IKK activation, IKK assay was performed by a method described previously [16]. Briefly, the IKK complex from whole-cell extracts (600 µg protein) was precipitated with antibody against IKKα followed by treatment with protein A/G-agarose beads. After 2 h of incubation, the beads were washed with lysis buffer and assayed in a kinase assay mixture containing 50 mM HEPES (pH 7.4), 20 mM MgCl_2_, 2 mM dithiothreitol, 20 µCi [γ^32^P]ATP,10 µM unlabeled ATP, and 2 µg of substrate glutathione S-transferase (GST)-IκBα (amino acids 1–54). After incubation at 30°C for 30 min, their action was terminated by boiling with SDS sample buffer for 5 min. Finally, the protein was resolved on 10% SDS-PAGE, the gel was dried, and the radioactive bands were visualized with a Phosphor Imager. To determine the total amounts of IKKα and IKKβ in each sample, the whole-cell protein was resolved on 10% SDS-PAGE, electrotransferred to a nitrocellulose membrane, and blotted with anti-IKKα or anti-IKKβ antibody.

### Trypan blue exclusion assay

Cells were harvested by treatment with 0.2% trypsin-EDTA, centrifuged, and suspended in one ml culture medium. Cell suspension was mixed with equal volume of 0.4% isotonic trypan blue solution. Total cell number and fraction of nonviable, dye-accumulating cells were counted after 2 min in hemocytometer under light microscope.

### Resorption pit assay

To examine the effect of cardamonin on bone resorption, a pit formation assay was carried out using calcium phosphate apatite-coated plates essentially following the manufacturer's protocol (BioCoat Osteologic Bone Cell Culture System; BD Biosciences).

### Statistical analysis

All experiments were carried out in triplicates and repeated twice. Data were expressed as mean ± SD and analyzed using Student's *t*-test. Statistical significance was considered when a *P* value was<0.05.

## Results

**Figure 1:**
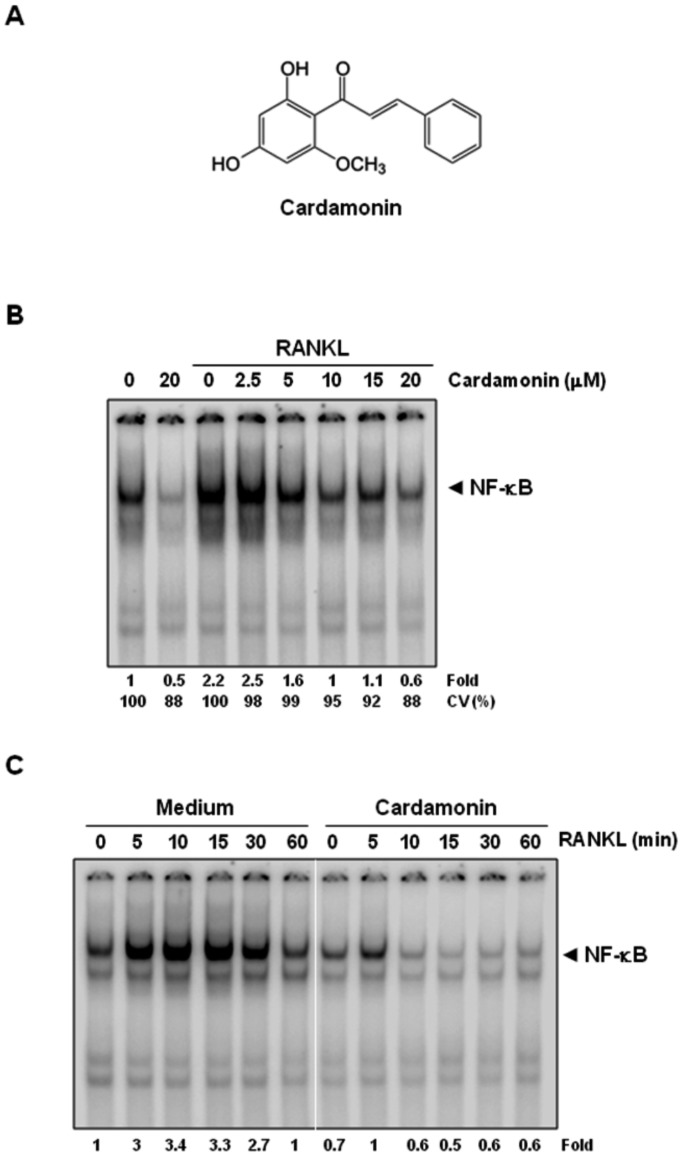
RANKL induces NF-κB activation and cardamonin inhibits it in a dose- and time-dependent manner. (**A**) The structure of cardamonin. (**B**) RAW 264.7 cells (1.5×10^6^/well) were incubated with different concentrations of cardamonin for 12 h, treated with 10 nM RANKL for 30 min, and examined for NF-κB activation by EMSA. Fold value is based on the value for medium (control), arbitrarily set at 1. (**C**) RAW 264.7 cells (1.5×10^6^/well) were incubated with 20 µM of cardamonin for 12 h, treated with 10 nM RANKL for the indicated times (min), and examined for NF-κB activation by EMSA. Fold value is based on the value for medium (control), arbitrarily set at 1. CV, cell viability.

The aim of this study was to investigate the effect of cardamonin (Fig. 1A) on RANKL signaling and on osteoclastogenesis. Whether cardamonin could modulate osteoclastogenesis induced by tumor cells was another focus of these studies. We used the RAW 264.7 cell (murine macrophage) system because it is a well-established model for osteoclastogenesis and contains no osteoblast/bone marrow stromal cells, thus allowing us to focus on RANKL signaling in pre-osteoclast cells [17].

### Cardamonin abrogates RANKL-induced NF-κB activation

To determine the concentration of cardamonin required to suppress RANKL-induced NF-κB activation, cells were pretreated with various concentrations of cardamonin and then exposed to RANKL. The chalcone almost completely suppressed NF-κB activation at 20 µM (Fig. 1B). Treatment of cells with 20 µM of cardamonin for 12 h had no effect on cell viability as determined by the trypan blue exclusion method.

To investigate whether cardamonin modulates RANKL-induced NF-κB activation in RAW 264.7 cells, cells were either pretreated with cardamonin for 12 h or left untreated and then exposed to RANKL for indicated times, nuclear extracts were prepared, and NF-κB activation was assayed by EMSA. As shown in Figure 1C, RANKL activated NF-κB in a time-dependent manner; however, cardamonin completely abrogated RANKL-induced NF-κB activation.

### Cardamonin inhibits RANKL-induced IκBα phosphorylation and degradation

**Figure 2:**
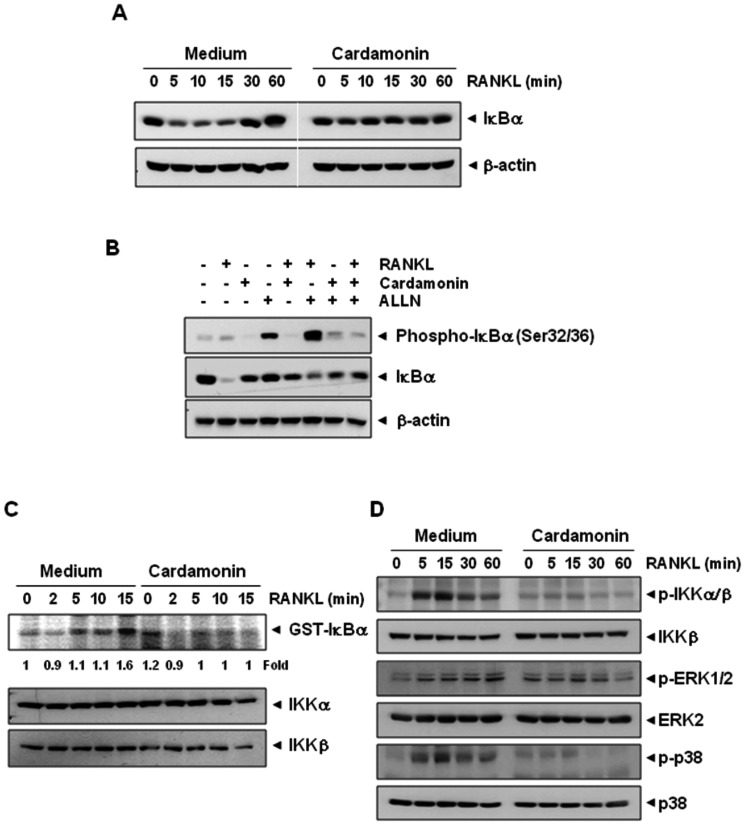
Cardamonin suppresses RANKL-induced IκBα degradation and phosphorylation through inhibition of IKK activity. (**A**) RAW 264.7 cells (1.5×10^6^/well) were incubated with 20 µM of cardamonin for 12 h and then treated with 10 nM RANKL for the indicated times (min). Cytoplasmic extracts were examined for IκBα degradation by western blot using an anti- IκBα antibody. Anti−β-actin was used as a loading control. (**B**) RAW 264.7 cells (1.5×10^6^/well) were pretreated with cardamonin (20 µM) for 12 h, then incubated with ALLN (50 µg/mL) for 30 min, and then treated with RANKL (10 nM) for 15 min. Cytoplasmic extracts were prepared and analyzed by western blot using phospho-IκBα antibody. The same membrane was reprobed with IκBα and β-actin antibody. (**C**) RAW 264.7 cells were pretreated with cardamonin (20 µM) for 12 h and then incubated with RANKL (10 nM) for the indicated times (min). Whole-cell extracts were immunoprecipitated using an antibody against IKKα and analyzed by an immune complex kinase assay using recombinant GST-IκBα as described in Materials and Methods. To examine the effect of cardamonin on the level of IKK proteins, whole-cell extracts were analyzed by western blot using anti-IKKα and anti-IKKβ antibodies. Values below the blot indicate fold change (GST-IκBα/IKKα) compared with control. Control was set as fold 1. (**D**) RAW 264.7 cells (1.5×10^6^/well) were pre-incubated with cardamonin for 12 h and then exposed to RANKL (10 nM) for the indicated times. Whole-cell extracts were analyzed by western blotting using relevant antibodies.

Because the proteolytic degradation of IκBα is required for translocation of NF-κB to the nucleus, we next examined whether suppression of NF-κB by cardamonin was due to inhibition of IκBα degradation. First, we examined NF-κB activation in the nucleus by EMSA (Fig. 1C) and IκBα degradation in the cytoplasm by western blot (Fig. 2A), after RANKL stimulation for various times. As shown in Figure 1C, RANKL activated NF-κB as early as within 5 min, and cardamonin completely inhibited this activation. In accordance with EMSA results, RANKL induced IκBα degradation in control cells within 5 min and returned to normal level within 60 min (Fig. 2A). On the other hand, cells pretreated with cardamonin showed no degradation of IκBα.

We next examined whether cardamonin affects the IκBα phosphorylation. Because phosphorylation, ubiquitination and proteasome mediated degradation of IκBα in response to RANKL is a rapid process, we used *N*-acetyl-leu-leu-norleucinal (ALLN), the proteasome inhibitor that prevents RANKL-induced IκBα degradation (Fig. 2B). When the cells were treated with RANKL alone, the intensity of the phospho-IκBα band was very weak (lane 2 in Fig. 2B). However, when cells were pretreated with ALLN before RANKL, an increase in phospho-IκBα was observed (lane 6 in Fig. 2B). When cells were treated with cardamonin before treatment with ALLN and RANKL, phosphorylation in IκBα was almost completely suppressed (lane 8 in Fig. 2B). Overall, these results suggest that phosphorylation and degradation of IκBα in response to RANKL alone is a rapid process and that cardamonin inhibits RANKL-induced NF-κB activation through suppression of IκBα degradation and phosphorylation.

### Cardamonin inhibits RANKL-induced IKK activation

Because cardamonin inhibits the phosphorylation and degradation of IκBα, we next investigated whether cardamonin affects the activity of IKK, which is needed for IκBα phosphorylation. As shown in Fig. 2C, RANKL induced IKK activation and cardamonin inhibited RANKL-induced IKK activation. We next examined whether the loss of IKK activity was due to the alteration of IKK protein expression; the protein levels of IKKα and IKKβ were determined by western blot analysis. Results in Figure 2C clearly show that neither RANKL nor cardamonin altered the expression of IKKα and IKKβ proteins.

### Cardamonin inhibits the RANKL-induced phosphorylation of IKK and signaling molecules linked with MAPK pathway

Because RANKL can induce IKK, and signaling molecules associated with MAPK pathway, we examined the effect of cardamonin on these signaling molecules. As shown in Figure 2D, RANKL induced the phosphorylation of IKK, ERK and p38 MAPK in osteoclast precursor RAW264.7 cells. However, pretreatment of cells with cardamonin significantly inhibited the RANKL-induced activation of these kinases.

### Cardamonin inhibits RANKL-induced osteoclastogenesis

**Figure 3:**
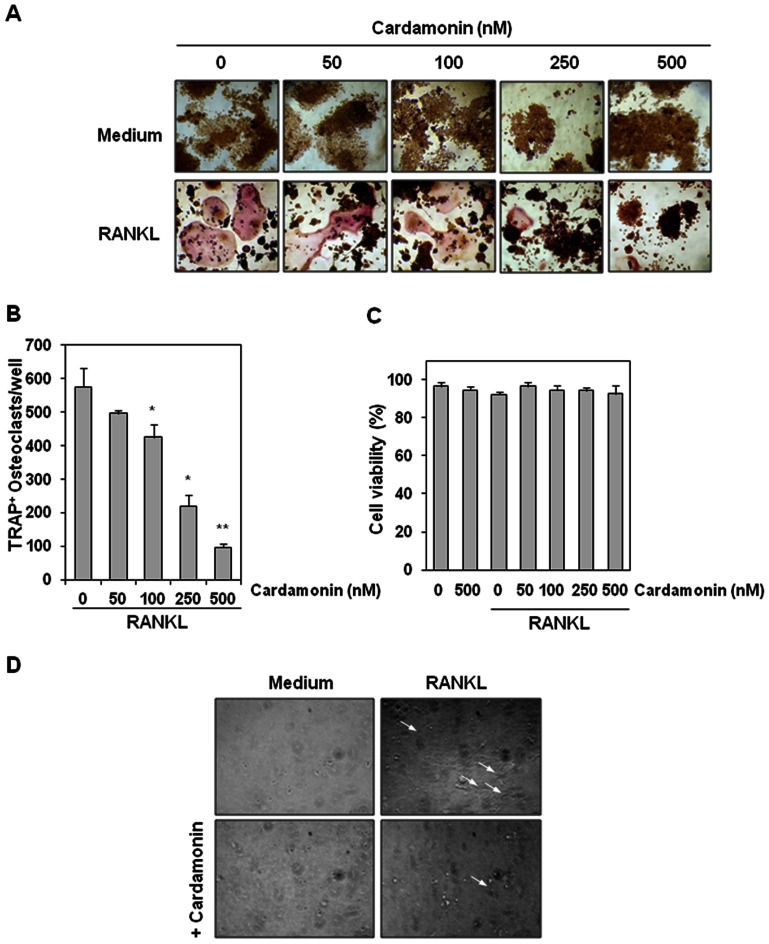
Cardamonin inhibits RANKL-induced osteoclastogenesis. (**A**) RAW 264.7 cells (5×10^3^/well) were incubated with cardamonin (500 nM) or RANKL (5 nM) alone, or with RANKL plus cardamonin (0, 50, 100, 250, 500 nM) for 5 days, and then stained for TRAP expression. TRAP-positive cells were photographed. Original magnification,×100. (**B**) Quantification of multinucleated osteoclasts (i.e., those containing three nuclei) after treatment with medium, RANKL (5 nM) alone, or RANKL plus cardamonin (0, 50, 100, 250, 500 nM) for 5 days. Values represent means ± SD. Data are representative of two independent experiments performed in triplicates; *, *P*<0.01, and **, *P*<0.05 *vs*. RANKL alone. (**C**) RAW 264.7 cells (5×10^3^ cells) were incubated with Indicated concentration of cardamonin for 12 h, and then treated with 5 nM RANKL for 5 days. To determine cell viability, cells were trypsinized, and then subjected to trypan blue exclusion assay. Data represent the means ± SD of triplicate samples. (**D**) RAW 264.7 cells (3×10^3^/well) were seeded into calcium phosphate apatite-coated plates, treated with cardamonin (500 nM) or RANKL (5 nM) alone, or with RANKL plus cardamonin. After 5 days, remaining cells were imaged under light microscope. Arrows indicate pit formation.

Whether cardamonin could inhibit RANKL-induced osteoclastogenesis was examined. Osteoclast precursor RAW 264.7 cells were treated with different concentrations of cardamonin in the presence of RANKL and allowed to differentiate into osteoclasts. As shown in Figure 3A, RANKL induced formation of osteoclasts in control cells. In contrast, differentiation into osteoclasts was significantly reduced in the presence of cardamonin. Moreover, the osteoclasts formation was decreased with increasing concentration of cardamonin (Fig. 3A and 3B). As little as 100 nM cardamonin had a significant effect on RANKL-induced osteoclast formation. Under these conditions, the viability of cells was not significantly affected (Fig. 3C).

We next examined whether cardamonin affects RANKL-induced bone resorption by measuring pit formation of RAW 264.7 cells. Results indicated that RANKL induced the pit formation, whereas cardamonin inhibited the pit formation (Fig. 3D). These results suggest that cardamonin can suppress bone resorption.

**Figure 4:**
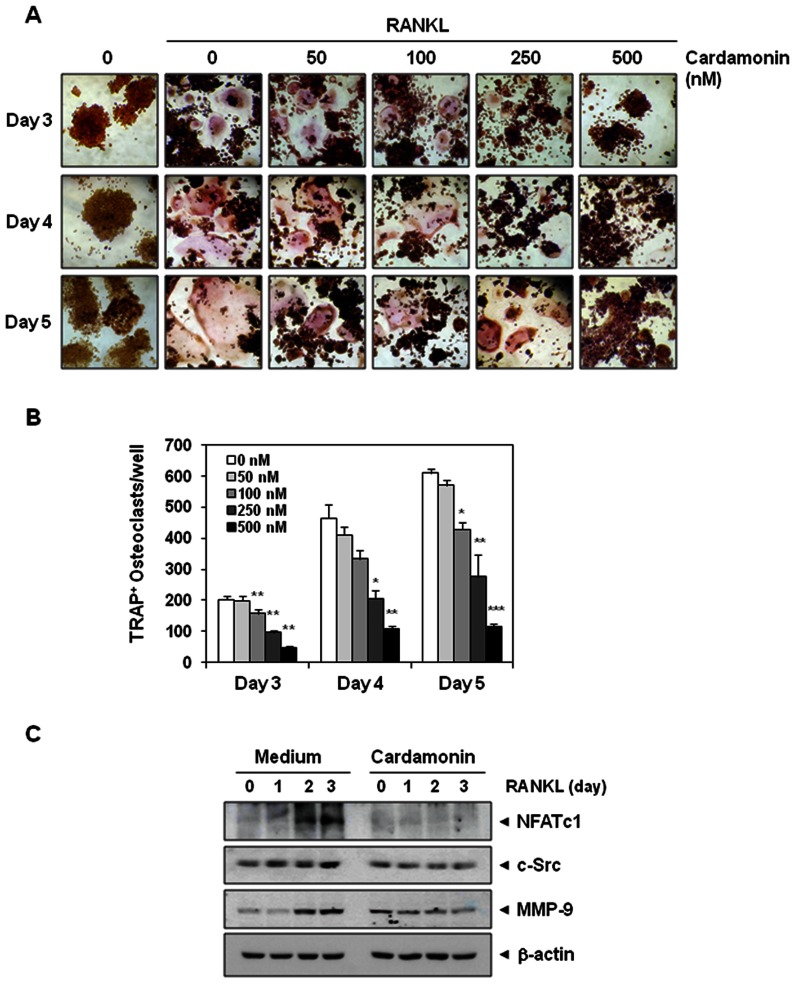
Cardamonin inhibits RANKL-induced osteoclastogenesis. (**A**) RAW 264.7 cells (5×10^3^/mL) were incubated with cardamonin (500 nM) or RANKL (5 nM) alone, or with RANKL plus cardamonin (0, 50, 100, 250, 500 nM), for 3, 4, or 5 days, and then stained for TRAP expression. TRAP-positive cells were photographed. Original magnification,×100. (**B**) Quantification of multinucleated osteoclasts (i.e., those containing three nuclei) after treatment with medium or RANKL plus cardamonin (0, 50, 100, 250, 500 nM) for 3, 4, or 5 days. Values represent means ± SD. Data are representative of two independent experiments performed in triplicates; *, *P*<0.05, **, *P*<0.01, and ***, *P*<0.001 *vs*. RANKL alone. (**C**) RAW 264.7 cells (0.7×10^6^/well) were pre-incubated with cardamonin (500 nM) for 12 h and then incubated with RANKL (5 nM) for the indicated days. Whole-cell extracts were analyzed by western blotting using relevant antibodies. Anti−β-actin was used as a loading control.

To determine whether cardamonin inhibits osteoclastogenesis in a time-dependent manner, RAW 264.7 cells were incubated with cardamonin for 3, 4, or 5 days and allowed to differentiate into osteoclasts by RANKL. Morphological observations clearly demonstrated that RAW 264.7 cells differentiated into osteoclasts after RANKL addition, and that cardamonin inhibited this differentiation (Fig. 4A). The extent of suppression was measured by counting the number of TRAP-positive osteoclasts per well (Fig. 4B). We observed that RANKL induced osteoclast differentiation in a time-dependent manner, with a maximum of TRAP-positive osteoclasts per well at day 5 (Fig. 4A and 4B). On the other hand, cardamonin decreased the number of TRAP-positive osteoclasts in a dose-dependent manner, with a strong inhibition at 500 nM at all days examined (Fig. 4B).

### Cardamonin inhibits RANKL-induced osteoclastogenesis markers

Whether cardamonin can suppress RANKL-induced osteoclastogenesis markers was investigated. Osteoclast precursor RAW 264.7 cells were treated with or without cardamonin in the presence of RANKL for 0, 1, 2, and 3 days. As shown in Figure 4C, RANKL up-regulated the expression of NFATc1, c-Src and MMP-9, whereas cardamonin significantly suppressed the expression of these osteoclastogenesis markers.

### Cardamonin acts at an early step in the pathway leading to RANKL-induced osteoclastogenesis

**Figure 5:**
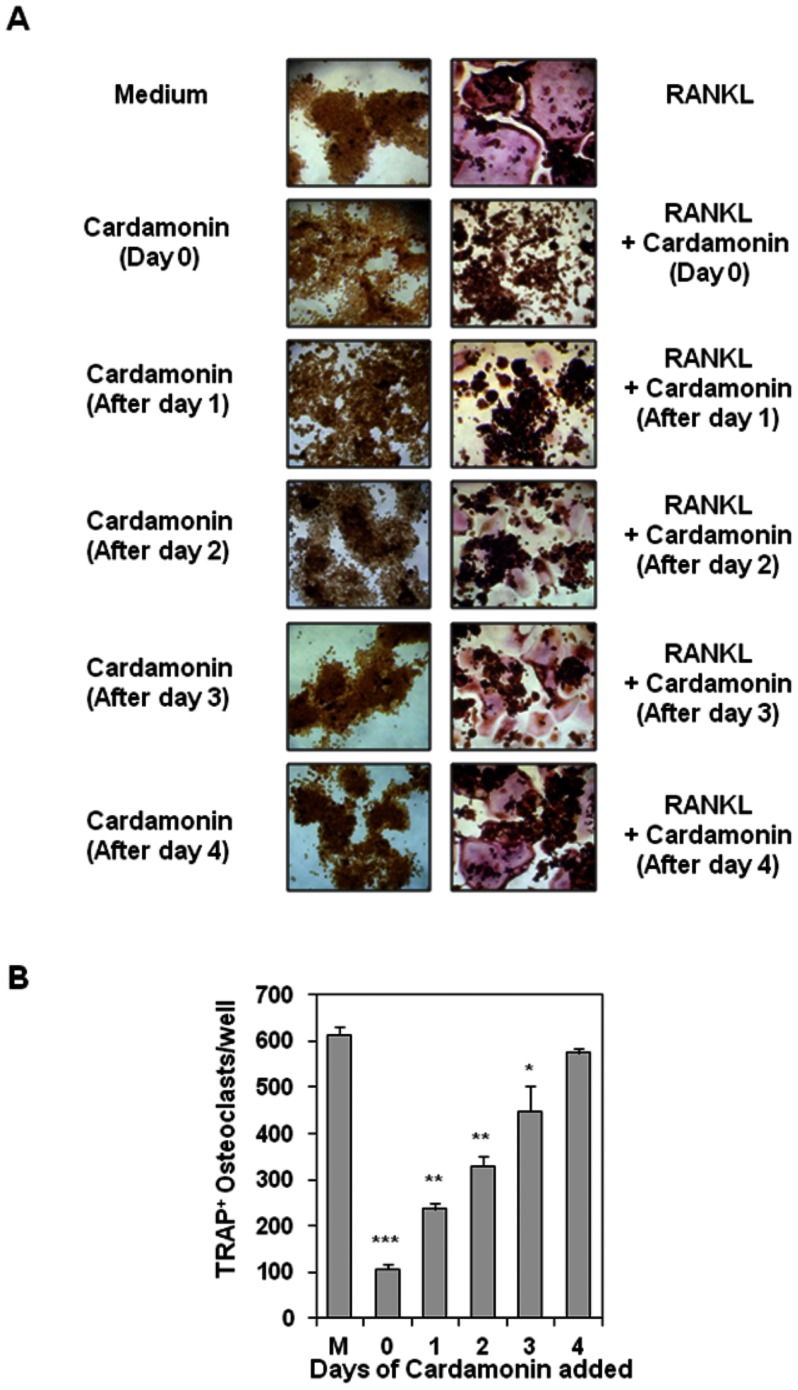
Cardamonin inhibits RANKL-induced osteoclastogenesis 24 h after stimulation. (**A**) RAW 264.7cells (5×10^3^/well) were incubated with RANKL (5 nM), cardamonin (500 nM), or both for the indicated times and stained for TRAP expression. (**B**) Multinucleated osteoclasts (i.e., those containing three nuclei) were counted. “M” stands for cells treated with medium alone. Values represent means ± SD. Data are representative of two independent experiments performed in triplicates; *, *P*<0.05, **, *P*<0.01, and ***, *P*<0.001 *vs*. medium alone.

It generally takes as long as 5 days for RAW 264.7 cells to differentiate into osteoclasts in presence of RANKL. To elucidate at what point in this pathway cardamonin acts, cells were treated initially with RANKL, cardamonin was added 1, 2, 3, and 4 days after RANKL addition, and then its effect on formation of osteoclast was measured (Fig. 5A). As determined by observation (Fig. 5A, right panel) and by counting of TRAP-positive osteoclasts per well (Fig. 5B), cardamonin markedly inhibited osteoclast formation when the cells were exposed to the compound for 1 or 2 days after RANKL stimulation. By days 3 and 4 after RANKL addition, however, osteoclast formation was no longer completely prevented by cardamonin (Fig. 5A and 5B), indicating that cardamonin probably acts at an early step in the osteoclast differentiation pathway.

### Cardamonin inhibits osteoclastogenesis induced by tumor cells

**Figure 6:**
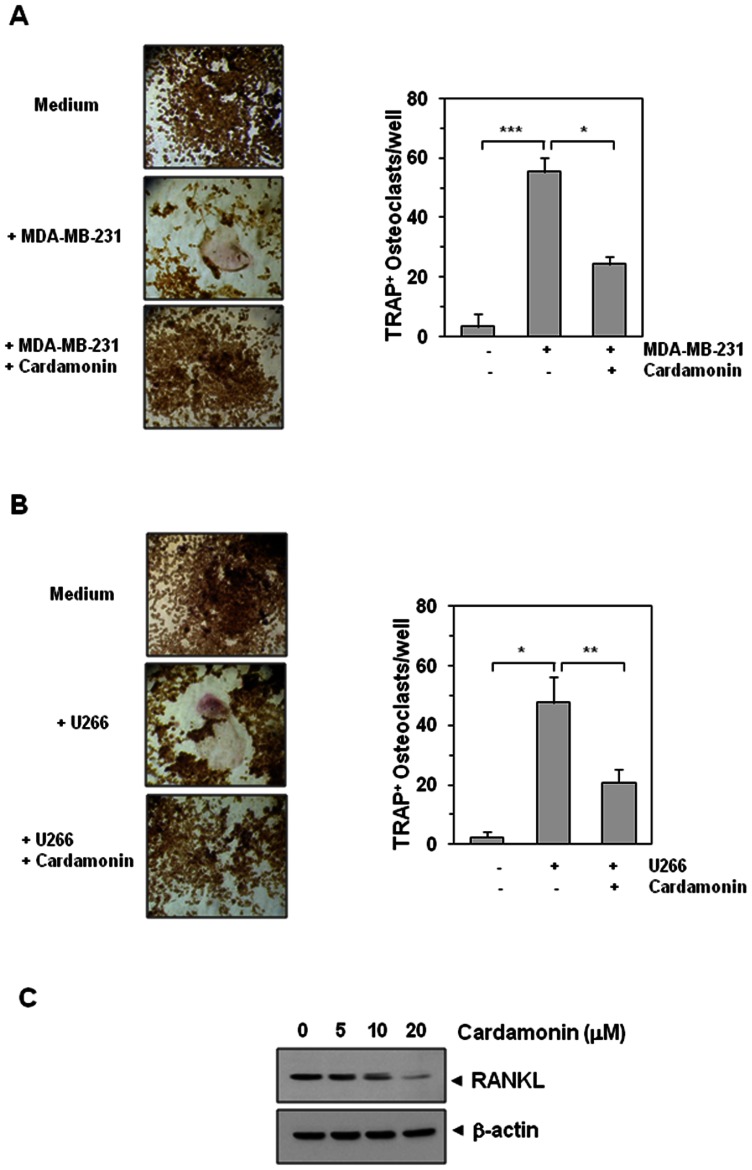
Cardamonin suppresses osteoclastogenesis induced by tumor cells. (**A**) RAW 264.7 cells (5×10^3^/well) were incubated in the presence of MDA-MB-231 cells (1×10^3^/well) and exposed to cardamonin (500 nM) for 5 days, and finally stained for TRAP expression. Multinucleated osteoclasts were counted (right panel). Values represent means ± SD. Data are representative of two independent experiments performed in triplicates; *, *P*<0.05, and ***, *P*<0.001. (**B**) RAW264.7 cells (5×10^3^/well) were incubated in the presence of U266 cells (1×10^3^/well) and exposed to cardamonin (500 nM) for 5 days, and finally stained for TRAP expression. Multinucleated osteoclasts were counted (right panel). Values represent means ± SD. Data are representative of two independent experiments performed in triplicates; *, *P*<0.05, and **, *P*<0.01. (**C**) U266 cells (1×10^6^/well) were incubated with indicated concentration of cardamonin for 24 h. Whole cell extracts were prepared and analyzed by western blotting using RANKL antibody. Anti−β-actin was used as a loading control.

Breast cancer [18] and multiple myeloma cells [19] are commonly linked with oteoclastogenesis. Whether cardamonin modulates osteoclastogenesis of RAW 264.7 cells induced by tumor cells was examined. Results indicated while both MDA-MB-231 breast cancer cells and U266 multiple myeloma cells induced RAW 264.7 cells to differentiate into osteoclasts, cardamonin suppressed the osteoclast formation (Fig. 6A and 6B). These results indicate that cardamonin suppress the osteoclastogenesis induced by tumor cells.

### Cardamonin suppresses RANKL expression in U266 cells

Because U266 cells induced osteoclstogenesis of RAW264.7 cells, whether U266 cells express RANKL was investigated. For this, the whole cell protein extract prepared from control and treated U266 cells were examined for RANKL expression. We found that U266 cells express RANKL and cardamonin suppressed the expression in a dose-dependent manner (Fig. 6C).

### Inhibition of NF-κB abrogates osteoclastogenesis

**Figure 7:**
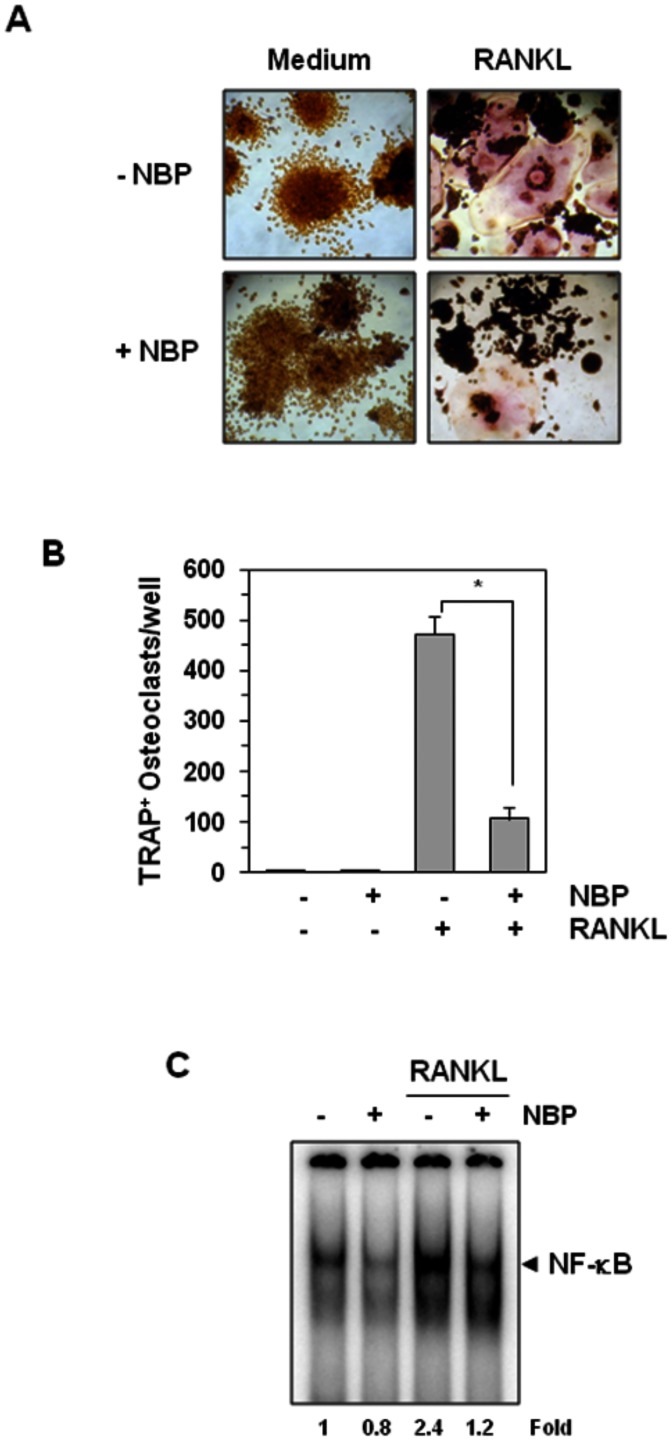
A peptide that targets the NEMO-binding domain inhibits RANKL-induced osteoclastogenesis. (**A**) RAW 264.7 cells (5×10^3^/well) were pretreated with 100 µM of the NEMO-binding domain peptide (NBP) for 2 h, medium was changed, and then RANKL (5 nM) was added for 5 days. (**B**) Multinucleated osteoclasts (i.e., those containing three nuclei) were counted. Values represent means ± SD. Data are representative of two independent experiments performed in triplicates; *, *P*<0.05. (**C**) RAW 264.7 cells (1.5×10^6^/well) were incubated with 100 µM of NBP for 2 h, and then incubated with 10 nM RANKL for 30 min and examined for NF-κB activation by EMSA. Fold value is based on the value for medium (control), arbitrarily set at 1.

Whether osteoclastogenesis induced by RANKL is due to activation of NF-κB by the cytokine, was also investigated using NF-κB specific inhibitor. NF-κB essential modulator (NEMO) binding peptide (NBP) is a peptide that binds to NEMO and thus blocks its activity to activate NF-κB. Our results in Figures 7A and 7B showed that RANKL induced osteoclastogenesis and NBP inhibited the differentiation. As shown in Fig. 7C, this peptide also blocked the RANKL-induced NF-κB activation.

## Discussion

Osteoclasts, which are responsible for bone resorption, are rare; each 1 mm^3^ of bone contains only two or three osteoclasts. Despite these small numbers, loss of function of osteoclasts or decrease in their number leads to osteosclerosis/osteopetrosis. On the other hand, an increase in their number or function induces bone osteoporosis, indicating that osteoclasts play a pivotal role in bone loss [20], [21]. Systemic hormones and cytokines provide the molecular cues that control osteoclastogenesis and thus maintain homeostasis [1]. RANKL has emerged as a major mediator of osteoclastogenesis. Major problems associated with aging and age-associated diseases such as arthritis, cancer, and other chronic inflammatory illnesses are disturbance of this balance. Thus safe, efficacious, and affordable compounds that can inhibit bone loss are needed. Cardamonin is one such compound that has been shown to suppress inflammatory pathways. The goal of this study was to investigate the effect of cardamonin, a bioactive chalcone, on RANKL-induced NF-κB activation and on osteoclastogenesis induced by both RANKL and tumor cells.

A number of inflammatory cytokines produced during different types of inflammation have a synergic role in osteoclastogenesis, include interleukins 1 and 6, TNF-α, and oncostatin M. These have been reported to stimulate osteoclastic differentiation and bone resorption [22], [23], [24]. Chemokines are chemotactic signals for monocytes that can facilitate the fusion of monocytes into multinucleated osteoclasts [25]. Our results indicate that RANKL activates NF-κB in osteoclast precursor cells through activation of IKK and subsequent IκBα phosphorylation and degradation. The RANKL-induced NF-κB activation signaling pathway differs from the TNF-induced pathway. For instance, NIK, which may function as an activator of IKKα, is necessary in RANKL-induced NF-κB activation [14]; however, it is dispensable for TNF-induced NF-κB activation [26]. Thus NIK-deficient osteoclast precursors have been reported to not respond to RANKL in an *in vitro* differentiation system devoid of osteoblasts [27]. We found that cardamonin inhibited RANKL-induced IKK activation, leading to suppression of NF-κB activation. That cardamonin inhibits RANKL-induced ERK1/2 and p38 activation indicates the multi-targeting activities of cardamonin.

Other studies have shown that IKKβ, but not IKKα, is a potent regulator of inflammation-induced bone loss and is required for osteoclastogenesis and inflammatory arthritis [28]. The study reported here is the first showing that cardamonin can suppress RANKL-induced IKK activation and consequently NF-κB activation. How cardamonin inhibits RANKL-induced IKK activation is not clear.

By recruiting the adapter proteins TRAF 2, 3, 5, and 6 and NIK, RANK activates NF-κB and the JNK, p38 MAPK, and p44/p42 MAPK signaling pathways [5], [13]. In addition to its role in inflammatory diseases, the NF-κB signaling pathway has been demonstrated to be a major mediator of bone loss [29]. It is already proven that NF-κB p50 and p52 expression are essential for the differentiation of RANK-expressing-osteoclast precursors into TRAP-positive osteoclasts in response to RANKL and other osteoclastogenic cytokines [30].

RANKL has been shown to play a major role in cancer-associated osteoclast differentiation. Furthermore, a series of electrolytes and degradative enzymes have been implicated in osteoclastogenesis, bone resorption, and calcium homeostasis [4], [31]. Mice deficient in the *rankl* gene have been shown to display severe osteopetrosis, stunted growth, defective tooth eruption, and osteoblasts that cannot support osteoclastogenesis [31]. Thus agents that can inhibit RANKL signaling have a great potential for inhibiting osteoclastogenesis.

Our results indicate that cardamonin effectively inhibits RANKL-induced osteoclastogenesis. A kinetic study indicated that cardamonin acts at an early step in the osteoclast differentiation process. To further confirm that inhibition of the NF-κB signaling pathway is responsible for arrest of the osteoclastogenesis process, we used a cell-permeable peptide that targets the NEMO-binding domain of the IKKα and IKKβ kinases and so prevents NF-κB activation. This NEMO-binding domain peptide has been shown to inhibit osteoclastogenesis *in vivo*, and also delayed the onset, lowered the incidence, and decreased the severity of rheumatoid arthritis [28]. Moreover, previous studies demonstrated that pharmacological or genetic inactivation of IKKα and/or IKKβ is sufficient for inhibition of osteoclastogenesis and prevention of inflammation and osteolytic bone loss [32], [33]. Our results show that the NF-κB inhibitor NEMO-binding domain peptide completely blocked RANKL-induced osteoclastogenesis in the same manner as cardamonin. Interestingly, the inhibitory effect of 100 µM of this peptide was as potent as 500 nM cardamonin, which suggests that cardamonin is 200 times more potent, at least *in vitro*. These findings indicate that cardamonin's inhibitory effect on osteoclastogenesis is probably specific to NF-κB inhibition. We found that the targets downstream to NF-κB activation pathway and known markers of osteoclastogenesis such as NFATc and MMP-9 were also suppressed by cardamonin treatment. It is likely that the reduction in NFATc and MMP-9 expression due to NF-κB inhibitory effects of cardamonin contribute to its anti-osteoclastogenic activities.

A major health problem today that affects over 350,000 patients in the United States annually is malignant tumors of skeleton as the primary site as well as metastatic bone lesions. Among them, 70% to 95% of multiple myeloma patients and up to 75% of patients with advanced breast cancer or prostate cancer develop bone metastasis. A major complication in metastatic breast cancer and multiple myeloma is osteoclast-mediated bone destruction [7], [34]. Breast cancers commonly cause osteolytic metastasis that depends on osteoclast-mediated bone resorption [35], but the mechanism responsible for this has not yet been clarified. We showed in this study that osteoclastogenesis induced by breast cancer cells is inhibited by cardamonin. Bone resorption is also associated with multiple myeloma [35], and we found that multiple myeloma cell-induced osteoclastogenesis was also suppressed by cardamonin. As IKK activation is known to accelerate the proliferation and metastasis of cancer cells [36], [37], its inhibitors, such as cardamonin, might have potential in the treatment of cancers that metastasize to the bone. These tumors have been shown to express RANKL [19], [38] and exhibit constitutive NF-κB activation [39], [40]. Thus, it can be concluded that these tumors activate osteoclastogenesis through RANKL expression.

The bisphosphonates are the only drugs now available for treatment of bone metastasis or cancer-related bone diseases. These drugs are highly toxic, however, and adverse effects such as renal impairment or osteonecrosis of the jaw have been reported [41]. An antibody to RANKL, denosumab (Prolia), has been approved recently for treatment of osteoporosis [42]. Cardamonin is derived from the seed of grass cardamom (*A. katsumadai* Hayata) and the fruit of black cardamom (*A. subulatum*), and should have minimum toxicity, as it is used routinely for traditional medicine [43], [44]. Thus cardamonin could be used in the treatment of secondary bone lesions associated with cancer and also nonmalignant diseases such as postmenopausal osteoporosis, Paget disease, and rheumatoid arthritis. Further studies are needed, however, to confirm whether cardamonin can suppress osteoclastogenesis *in vivo*.
